# Radiographic Analysis of Pedicle Screw Retractor‐Assisted Transforaminal Lumbar Interbody Fusion for Single‐Segment Spondylolisthesis in Adults: A Retrospective Study and Technical Note

**DOI:** 10.1111/os.13441

**Published:** 2022-08-18

**Authors:** Hongwei Xie, Ziyu Ouyang, Hua Zhang

**Affiliations:** ^1^ Department of Orthopaedic Surgery, the Second Affiliated Hospital Zhejiang University School of Medicine Hangzhou PR China; ^2^ Orthopaedics Research Institute Zhejiang University Hangzhou City Zhejiang Province PR China; ^3^ Key Laboratory of Motor System Disease Research and Precision Therapy of Zhejiang Province Hangzhou City Zhejiang Province PR China; ^4^ Clinical Research Center of Motor System Disease of Zhejiang Province Hangzhou City Zhejiang Province PR China

**Keywords:** spinal disease, spondylolisthesis, surgical instruments, zygapophyseal joint

## Abstract

**Objectives:**

The objective of this study was to introduce a retractor that can be temporarily installed on unilateral pedicle screws to achieve distraction‐reduction and nerve root protection, and to analyze the efficacy and safety of retractor‐assisted transforaminal lumbar interbody fusion (TLIF) in the treatment of lumbar spondylolisthesis.

**Methods:**

This was a retrospective study of 125 patients who underwent retractor‐assisted TLIF for single‐segment spondylolisthesis from November 2017 to February 2021. Based on morphology, patients were divided into degenerative (*N* = 66) and isthmic groups (*N* = 59). Differences in demographics and preoperative characteristics between the groups were analyzed using the independent samples *t*‐test and *χ* ^2^ test. Changes in radiographic parameters (disc height, foramen height, spondylolisthesis degree, slippage length, and segmental lordosis) before and after surgery were compared using the paired samples *t*‐test. Logistic regression analysis was performed to analyze the relationship between facet joint angle (FJA) and degenerative lumbar spondylolisthesis (DLS).

**Results:**

Unilateral screw retractor‐assisted TLIF significantly corrected spondylolisthesis and improved disc height and segmental lordosis (*p* < 0.05). There was no significant difference in foramen height between the two sides before and after operation (pre: 15.81 ± 3.58 mm *vs* 15.69 ± 3.68 mm, *p* = 0.599; post: 18.65 ± 2.31 mm *vs* 18.74 ± 2.26 mm, *p* = 0.516). The degree of spondylolisthesis in the DLS group before surgery was significantly lower than that in the isthmic spondylolisthesis group (17.70 ± 5.62% *vs* 25.18 ± 9.73%, *p* < 0.001), whereas a similar degree of correction could be achieved after surgery (5.91 ± 3.12% *vs* 7.16 ± 5.69%, *p* = 0.135). FJAs from L3/4 to L5/S1 were significantly smaller in patients with DLS than those in with isthmic spondylolisthesis (*p* < 0.05). Patients with facet sagittalization were more likely to have DLS (*β*: −0.101, odds ratio [OR]:0.904, 95% confidence interval [CI]: 0.874–0.934, *p* < 0.001), while the cut‐off FJA of L4/5 for predicting L4 spondylolisthesis was 53.19.

**Conclusions:**

Pedicle screw retractor‐assisted TLIF is effective and safe in treating both degenerative and isthmic lumbar spondylolisthesis. The unilateral retractor has the capacity to maintain the disc height achieved by paddle distractors, which optimizes the nerve protection and distractor placement. Patients with an FJA on L4/5 <53.19 were more likely to have DLS.

## Graphical abstract

The pedicle screw retractor can be temporarily installed on unilateral pedicle screws to provide traction to expand the intervertebral space, allowing for more operation space and nerve protection for transforaminal lumbar interbody fusion (TLIF). Furthermore, the retractor had the capacity to maintain the disc height achieved by paddle distractors, allowing for the progressive and stable distraction of the intervertebral space by exchanging distractors. Short‐term radiographic outcomes have shown that surgical reduction was sufficient and stable, with no significant complications such as endplate injury, screw loosening, or iatrogenic scoliosis, demonstrating that this instrument was safe and effective.

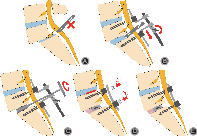

## Introduction

Lumbar spondylolisthesis (LS) is defined as the anterior or posterior displacement of one vertebral segment in reference to the segment beneath it. In 1976, Wiltse^1^ proposed classifying the LS into five categories—dysplastic, isthmic, degenerative, traumatic, and pathologic—depending on both etiological and anatomical factors. Among these, degenerative lumbar spondylolisthesis (DLS) and isthmic lumbar spondylolisthesis (ILS) are the most common.^2^ Spondylolysis is characterized by bone defects at the vertebral arch, which primarily occurs in adolescents and elite athletes.^3^, ^4^ The defect of the pars interarticularis is caused by repetitive stress loading on the lumbar spine during extension and rotation, and the spondylolysis with bilateral defects is more likely to progress to ILS.^5^ DLS, in contrast to ILS, refers to LS with a complete vertebral arch and occurs primarily in patients over the age of 50,^6^ with few cases diagnosed prior to this age. DLS is widely accepted to be caused primarily by the progressive degeneration of intervertebral discs and facet joints with aging.^7^ Moreover, a large‐scale epidemiological study based on the Chinese population^6^ revealed that the female‐to‐male prevalence ratio was approximately 1.3:1. Researchers believe that gestation, menopause, and poorer muscular stabilization make the progression of DLS faster in female patients than in male patients.

LS may give rise to lumbar spinal stenosis and neurologic compression. Consequently, this could lead to back or leg pain and neurological symptoms, negatively compacting quality of life. When conservative treatment fails, surgical interventions including decompression, reduction, and fixation are necessary.

Transforaminal lumbar interbody fusion (TLIF) has been widely used to treat LS associated with neurological symptoms^8^ and has been proven to achieve satisfactory clinical outcomes.^9^, ^10^ Nevertheless, LS is often accompanied by varying degrees of disc collapse and foraminal stenosis, which may hinder the surgeon's operation in the intervertebral space. Several techniques and instruments are available to distract the vertebrae and assist the interbody fusion. The first solution is the utilization of a distractor into the intervertebral space. Sears *et al*.^11^ used this surgical instrument in posterior lumbar interbody fusion (PLIF). The paddle‐shaped interbody distractor was inserted into the intervertebral space and rotated to 90°, with the width of the “paddle” as the distraction height. Simultaneously, the slipped vertebral body could be reduced by approximately 50% using the tension of the anterior longitudinal ligament. The ball‐bearing slide‐type interbody distractor proposed by Li *et al*.^12^ can produce greater longitudinally sustained force and backward traction to the vertebral bodies, thus completing the transverse and longitudinal reduction of LS. However, when the intervertebral space is too narrow to restore height directly by placing paddle distractors, surgeons consider indirect distraction and reduction of the vertebral bodies by distracting the pedicle screws. The SOCON® spondylolisthesis reduction assembly adopted by Floman *et al*.^13^ can pull the screws longitudinally and horizontally. Moreover, Tumialan *et al*.^14^ developed a provisional ipsilateral expandable rod used in minimally invasive TLIF (MIS‐TLIF), which can be installed on the external area of two fixation screws on the same side, with the maximum screw spacing being able to be extended to 42 mm.

However, there is a certain risk of endplate injury when using an interbody distractor or a rimer to expand the intervertebral space alone,^15^ which may lead to postoperative cage subsidence^16^ or even cage retropulsion,^17^ resulting in poor clinical outcomes. Therefore, in order to avoid this situation, it may be feasible to reduce the stress applied to the endplate during disc resection and fusion cage implantation. Meanwhile, another issue is worth addressing, unilateral distraction may not be captured equally by the opposite side, resulting in coronal imbalance and an increased risk of iatrogenic scoliosis,^18^ which limit the use of TLIF auxiliary instruments.

Considering these factors, we adopted screw retractor‐assisted TLIF to overcome the current limitations in the treatment of spondylolisthesis. This study aimed to: (i) introduce the design and instructions of the pedicle screw retractor; (ii) investigate the efficacy and safety of screw retractor‐assisted TLIF for adult LS; (iii) explore the potential relationship between FJAs and DLS.

## Materials and Methods

### Study Design

This retrospective study was conducted from November 2017 to February 2021 at the Second Affiliated Hospital, Zhejiang University School of Medicine. A total of 125 adult patients who underwent TLIF with the assistance of the pedicle screw retractor were enrolled in this study. All surgeries were performed by one senior spine surgeon. Patients were divided into two groups according to etiology: DLS (*n* = 66) and ILS (*n* = 59).

### Inclusion and Exclusion Criteria

Single‐segment LS, diagnosed by computed tomography (CT) scan, was defined as only one vertebral body slipping forward at a minimum of 3 mm relative to the inferior vertebral body. Adult patients included in the study fulfilled the following criteria: (i) preoperative symptoms including claudication with low back pain or leg pain, wherein conservative treatment was ineffective; and (ii) CT and X‐ray examinations performed before and after operation, wherein the image quality was qualified.

The exclusion criteria were as follows: multi‐segment spondylolisthesis or requiring surgical treatment for adjacent segments, spinal fractures, traumatic spondylolisthesis, spinal tumors, spinal tuberculosis, soft‐tissue injuries, soft‐tissue infections, congenital malformations of the spine, history of lumbar interbody fusion surgery, and low‐quality radiologic data.

### Screw Pedicle Retractor: Instrument Design

**FIGURE 1:**
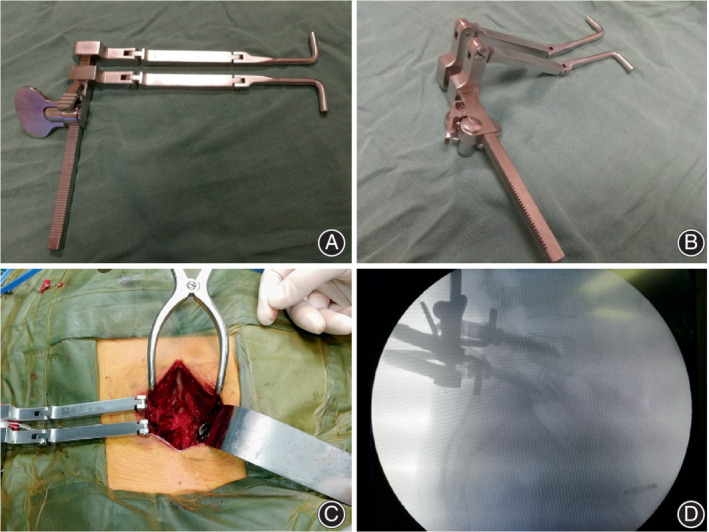
Illustration of pedicle screw retractor. (A, B) Photograph of pedicle screw retractor. (C) Intraoperative photograph: retractor is installed on two unilateral adjacent screws and is locked by nuts. (D) Retractor was seen on the intraoperative lateral radiograph after the insertion of a kidney‐shaped cage

This pedicle screw retractor (Type L Lumbar Retractor, Qingniu, Jiangsu, China) consists of two L‐shaped pedicle screw connecting rods and a distraction guide fixture (Figure 1). The screw connecting rods can be fixed to the adjacent pedicle screws by locking the screw cap. The surgeon can increase the distance between the two connecting rods by rotating the distraction knob, so as to drive the pedicle screws to distract the vertebral bodies. The check ratchet on the distraction guide fixture is used to lock the screw connecting rods. This can capture and maintain the intervertebral height achieved by rotating the interbody distractors.

### Technical Description of Surgery

**FIGURE 2:**
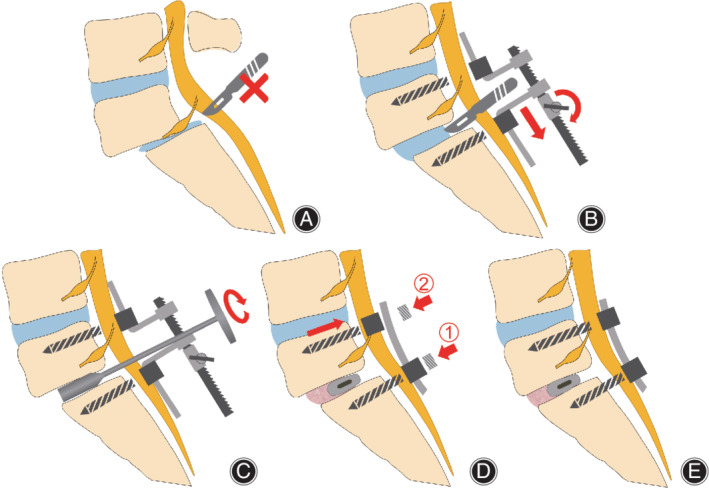
Schematic illustration of the key procedure in retractor‐assisted TLIF. (A) The intervertebral disc was covered by nerve root in abnormal position. (B) Installing the screw retractor to complete discectomy. (C) After the interbody distractor was inserted and rotated to 90°, the spondylolisthesis was initially reduced. (D, E) Locking the screws to the rod from the caudal side to the cephalad side to complete the reduction

The patient was placed in a prone position on the operating table under general anesthesia. The skin was cut at the posterior midline of the waist, and the spinous process, supraspinous and interspinous ligaments were retained, and the paraspinal muscles were peeled off to the outer edge of the superior articular process along the periosteum of both sides of the spinous process. Lateral fluoroscopy was performed to determine the spondylolisthesis and its inferior vertebral body. The cortical bone of the mastoid process was removed using a rongeur as the entry point, and a long‐tail universal pedicle screw with appropriate diameter and length was inserted bilaterally. After lateral fluoroscopy confirmed that the pedicle screws were in a good position, the screw retractor was installed on the two screws on the decompression side (Figure 2B). Total or semi‐laminectomy was performed on the medial edge of the articular process to remove the hypertrophic ligamentum flavum and hyperplastic tissue for full decompression. In this procedure, when the knob is properly rotated to enlarge the intervertebral space, the posterior lateral edge of the intervertebral disc can be clearly exposed. After cutting the fibrous annulus, the endplate curette and the nucleus pulposus forceps were used to remove the disc space contents. The interbody distractor was inserted into the intervertebral space and rotated to 90° to restore the disc height, thus, the spondylolisthesis was preliminarily reduced.^11^ The interbody distractor was replaced gradually from small to large, and the distraction knob was adjusted to lock the retractor before each replacement. The procedure was repeated until the tension of annulus fibrosus reached its maximum (Figure 2C).

Implant and compact synthetic bone substitute in the front 1/3 of the disc space and one kidney‐shaped interbody cage filled with autologous morselized bone grafts from posterior elements were inserted obliquely. After the cage entered the intervertebral space entirely, a bone hammer was used to hit the cage to make it enter the midline position of the intervertebral space obliquely. The pedicle screws were connected by a pre‐bent longitudinal rod, and the nuts were locked from the caudal side to the cephalad side (Figure 2D). The posterior translation force of the cephalad screw was used to draw the vertebrae and the pedicle screw to the rod to complete the reduction of spondylolisthesis and restore spinal alignment.^19^ The key procedures of the surgical technique are shown in Figure 2. A subfascial drainage tube was placed on the decompression side, and the incision was sutured layer by layer.

The drainage tube was removed 48 h after surgery, or when the drainage volume was less than 50 ml within 8 h. A brace was placed on the patients for 1 month postoperatively.

### Radiographic Assessment

In this retrospective single‐center study, all patients were evaluated under identical conditions with CT scans before and after surgery. All the measurements were performed with a picture archiving and communication system (PACS). Follow‐up was performed at 2 days, 1 and 3 months after surgery. Radiographs were taken of the lumbar spine in the frontal and lateral positions, recording complications and fusion conditions. Endplate injury was defined as the iatrogenic damage to the cortical endplate detected on the postoperative sagittal or coronal CT scans, which was absent preoperatively. All the radiographic parameters were measured by two qualified surgeons blinded to clinical and radiographic information.

### Clinical and Radiographic Parameters

The age of each patient at the time of admission, body mass index (BMI), operation time, estimated blood loss, and length of hospital stay were recorded.

The facet joint angle (FJA), which represented the orientation of the facet joint (a line connecting the anteromedial and posteromedial edges of the superior articular facet), was measured as described by Chadha *et al*.^20^ The angle between the two facet lines bilaterally was the FJA. The FJA from L3/4 to L5/S1 were measured.

**FIGURE 3:**
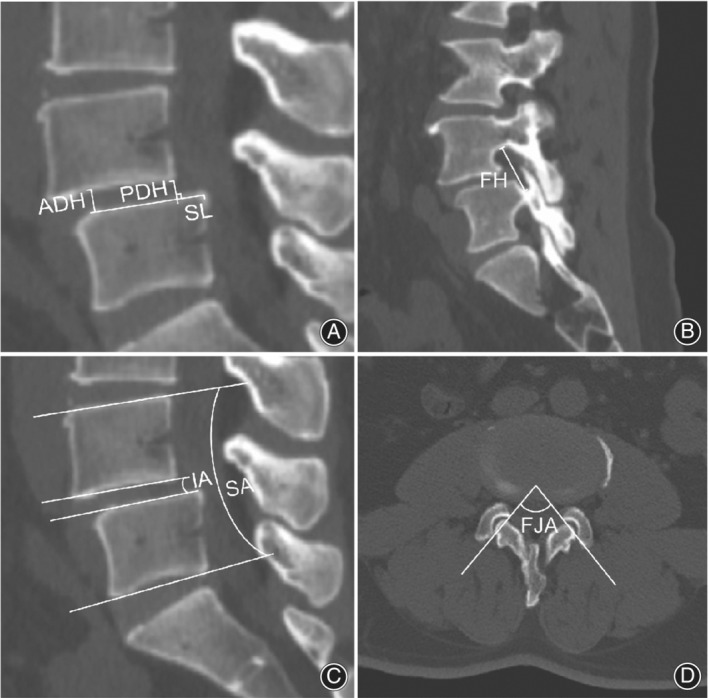
Measurement of radiographic parameters. (A) Anterior disc height (ADH), posterior disc height (PDH), DH = (ADH + PDH)/2, slippage length (SL). (B) Foramen height (FH). (C) Segmental angle (SA), intervertebral angle (IA). (D) Facet joint angle (FJA)

Other radiographic parameters measured by CT scan in the supine position include slippage length (SL), spondylolisthesis degree (SD), segmental angle (SA), intervertebral angle (IA), disc height (DH), and foramen height (FH), and were defined as follows (Figure 3A–D):

- SL: The sagittal CT image crossing the midline of the spinous process was selected. This was the distance that the posterior edge of the inferior endplate of the spondylolisthesis moved forward relative to the posterior edge of the upper endplate of the lower vertebra, as defined in the Meyerding grading system^21^.
- SD: The ratio of SL to anteroposterior diameter of the lower vertebral body × 100%.
- DH: The average distance between the upper endplate and the lower endplate of the intervertebral disc at the anterior and posterior edges.
- FH: The maximum distance between the inferior margin of the pedicle of the superior vertebrae and the superior margin of the pedicle of the inferior vertebral body. It includes the left FH (LFH) and the right FH (RFH).
- SA: The angle between the superior endplate of the slipped vertebral body and the inferior endplate of the inferior vertebral body in the sagittal plane.
- IA: The angle between the inferior endplate of the slipped vertebral body and the superior endplate of the inferior vertebral body.

### Statistical Analysis

All data analyses were performed using SPSS for Windows software (IBM SPSS Statistics 19, SPSS). All data were expressed as the mean ± standard deviation. Differences before and after surgery in DH, FH, IA, SA, SD, and SL were analyzed by the paired samples *t*‐test. The difference in demographic and baseline data between two groups was analyzed using the *χ* ^2^ test and the independent‐samples *t*‐test. Hypothetical risk factors were analyzed using logistic regression. Statistical significance was set at *p* < 0.05.

## Results

### Demographic Data of Included Patients

The ILS group included 59 patients (22 male patients, 37 female patients) with an average age at surgery of 53.03 ± 10.20 years. The DLS group included 66 patients (21 male patients, 45 female patients) with an average age at surgery of 63.07 ± 8.57 years. In patients with single‐segment ILS, 39.0% of spondylolisthesis occurred at L4 and 61.0% at L5. It should also be noted that spondylolisthesis at L5 was caused by spondylolysis, while single‐segment DLS occurred exclusively at L4. There were no significant differences in gender, BMI, operation time, estimated blood loss, and hospital stay between the two groups. The baseline demographic data of the patients in the study are illustrated in Table 1.

**TABLE 1:** Demographic baseline data

	Total	Spondylolisthesis		*t*‐value	*p*‐value
		Degenerative	Isthmic		
Number	125	66	59		
Gender					0.520
Male	43	21	22		
Female	82	45	37		
Spondylolisthesis degree					<0.001^a^
Grade I	81	57	24		
Grade II	44	9	35		
Level of fusion					
L4–L5	89	66	23		
L5–S1	36	0	36		
Age (years)	58.34 ± 10.61	63.08 ± 8.57	53.03 ± 10.20	5.978	<0.001^a^
BMI (kg/m^2^)	24.28 ± 3.37	24.47 ± 3.34	24.17 ± 3.43	0.337	0.736
Estimated blood loss (ml)	107.20 ± 60.14	105.30 ± 56.25	109.32 ± 64.64	−0.372	0.711
Operation time (minutes)	103.17 ± 23.422	99.94 ± 22.42	106.78 ± 24.17	−1.641	0.103
Hospital stay (days)	8.72 ± 3.22	9.15 ± 3.47	8.24 ± 2.87	−1.594	0.114

Abbreviation: BMI, body mass index

^a^ Statistically significant if *p* < 0.05.

### Changes in Various Radiographic Parameters After TLIF

The degree of spondylolisthesis was significantly decreased from 21.23 ± 8.65% before the operation regimen to 6.50 ± 4.54% after (*p* < 0.001). Table 2 displays the radiographic changes in the intervertebral space and foramen before and after surgery. On average, spondylolisthesis was corrected by 4.91 ± 2.46 mm (pre: 7.11 ± 2.90 mm *vs* post: 2.20 ± 1.56 mm, *p* < 0.001). DH and FH were significantly improved compared to those before surgery (*p* < 0.001).

**TABLE 2:** Radiographic outcomes of TLIF on treating spondylolisthesis

	Pre‐	Post‐	*t*‐value	*p*‐value
DH (mm)	7.76 ± 2.59	10.61 ± 1.73	−12.982	<0.001^a^
SL (mm)	7.11 ± 2.90	2.20 ± 1.56	22.458	<0.001^a^
SD (%)	21.23 ± 8.65	6.50 ± 4.54	22.096	<0.001^a^
LFH (mm)	15.69 ± 3.68	18.74 ± 2.26	−8.458	<0.001^a^
RFH (mm)	15.81 ± 3.58 (*p* = 0.599)	18.65 ± 2.31 (*p* = 0.516)	−7.705	<0.001^a^
IA (°)				
L4/5	5.27 ± 3.26	6.58 ± 2.77	−4.670	<0.001^a^
L5/S1	7.53 ± 4.40	9.90 ± 4.04	−3.415	0.002^a^
SA (°)				
L4/5	18.96 ± 5.88	19.89 ± 6.11	−2.915	0.005^a^
L5/S1	29.10 ± 5.69	31.43 ± 5.89	−3.207	0.003^a^

Abbreviations: DH, disc height; IA, intervertebral angle; LFH, left foramen height; RFH, right foramen height; SA, segmental angle; SD, spondylolisthesis degree; SL, slip length

^a^ Statistically significant if *p* < 0.05.

No significant differences were observed for FH between the two sides before and after the surgery (pre: 15.81 ± 3.58 mm *vs* 15.69 ± 3.68 mm, *p* = 0.599; post: 18.65 ± 2.31 mm *vs* 18.74 ± 2.26 mm, *p* = 0.516). IA and SA were significantly increased in patients with L4 or L5 spondylolisthesis compared to those before surgery (*p* < 0.05).

### Comparisons between ILS Group and DLS Group

Comparisons of the reduction between the two groups, ILS and DLS, are shown in Table 3. The preoperative length and degree of spondylolisthesis in the ILS group were significantly greater than those in the DLS group (8.37 ± 3.29 mm *vs* 5.98 ± 1.90 mm, *p* < 0.001; 25.18 ± 9.73% *vs* 17.70 ± 5.62%, *p* < 0.001). However, the postoperative length and degree of spondylolisthesis were similar in both groups (2.41 ± 1.94 mm *vs* 2.02 ± 1.09 mm, *p* = 0.174; 7.16 ± 5.69% *vs* 5.91 ± 3.12%, *p* = 0.135). The pre‐ and postoperative DHs in the ILS group were 6.37 ± 2.72 mm and 10.03 ± 1.92 mm, respectively. Meanwhile, the pre‐ and postoperative DHs in the DLS group were 9.01 ± 1.71 mm and 11.13 ± 1.36 mm, respectively. The DH restoration was 3.66 ± 2.03 mm and 2.12 ± 1.19 mm, respectively.

**TABLE 3:** Differences in radiographic outcomes between groups (*n* = 125, 66 DLS/59 ILS)

	Degenerative	Isthmic	*t*‐value	*p*‐value
SL (mm)				
Pre‐	5.98 ± 1.90	8.37 ± 3.29	−4.893	<0.001^a^
Post‐	2.02 ± 1.09	2.41 ± 1.94	−1.370	0.174
△SL	3.97 ± 1.88	5.97 ± 2.62	−4.844	<0.001^a^
SD (%)				
Pre‐	17.70 ± 5.62	25.18 ± 9.73	−5.184	<0.001^a^
Post‐	5.91 ± 3.12	7.16 ± 5.69	−1.510	0.135
△SD	11.80 ± 5.78	18.02 ± 7.89	−4.976	<0.001^a^
DH (mm)				
Pre‐	9.01 ± 1.71	6.37 ± 2.72	6.407	<0.001^a^
Post‐	11.13 ± 1.36	10.03 ± 1.92	3.644	<0.001^a^
△DH	2.12 ± 1.19	3.66 ± 2.03	−5.098	<0.001^a^
FJA (°)				
L3/4	60.00 ± 17.63	69.42 ± 12.37	−3.486	0.001^a^
L4/5	38.57 ± 20.01	81.23 ± 17.77	−12.540	<0.001^a^
L5/S1	84.56 ± 23.16	93.59 ± 18.01	−2.413	0.017^a^

Abbreviations: DH, disc height; FJA, facet joint angle; SD, spondylolisthesis degree; SL, slip length

^a^ Statistically significant if *p* < 0.05.

### Correlation between the FJA and DLS

The average FJA of L3/4, L4/5, and L5/S1 in DLS group was 60.00 ± 17.63°, 38.57 ± 20.01, and 84.56 ± 23.16, respectively. Meanwhile, the FJA of each segment in the ILS group was 69.42 ± 12.37, 81.23 ± 17.77, and 93.59 ± 18.01, respectively. The FJAs in the ILS group were significantly greater than those in the DLS group (*p* < 0.05) and showed a trend of gradual increase. In the DLS group, the FJA of L4/5 was significantly smaller than that of L3/4 and L5/S1 (*p* < 0.001), which was the opposite to the trend observed in the ILS group.

**FIGURE 4:**
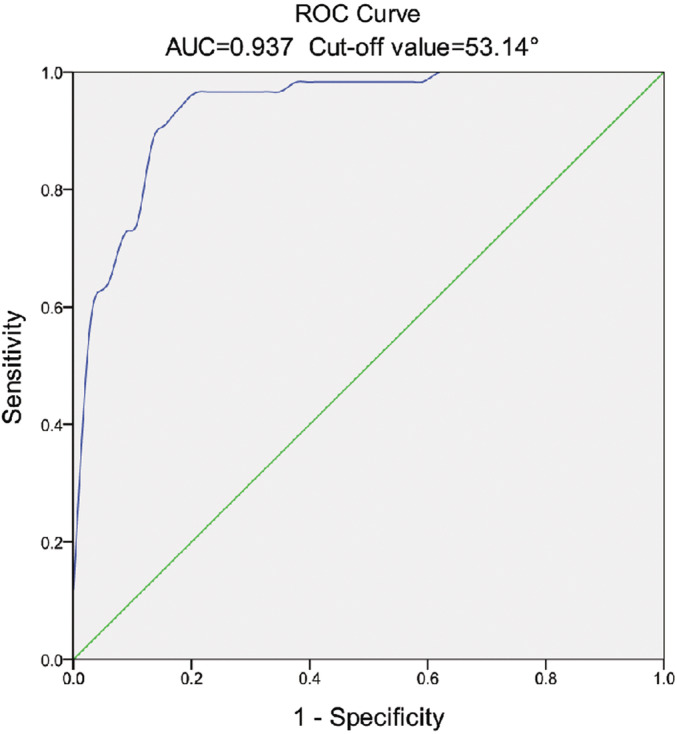
ROC curve of L4/5 FJA predicting L4 DLS. The cut‐off value of L4/5 FJA is 53.14°, corresponding to an AUC of 0.937. AUC, area under curve; DLS, degenerative lumbar spondylolisthesis; FJA, facet joint angle; ROC, receiver operator curve

Logistic regression analysis demonstrated that smaller FJAs were significant predictors for DLS, including L3/4 (*β*: −0.042, odds ratio [OR]: 0.959, 95% confidence interval [CI]: 0.934–0.984, *p* = 0.002), L4/5 (*β*: −0.101, OR: 0.904, 95% CI: 0.874–0.934, *p* < 0.001) and L5/S1 (*β*: −0.021, OR: 0.979, 95% CI: 0.961–0.997, *p* = 0.017) (Table 4). The receiver operating characteristic curve of L4/5 FJA for predicting the probability of DLS is shown in Figure 4. The cut‐off value of L4/5 FJA for predicting DLS was 53.14, corresponding to an area under the curve (AUC) of 0.937 (95% CI: 0.895–0.978, *p* < 0.001).

**TABLE 4:** Logistic regression analysis of FJA predicting DLS

	*β*	Comparison	Odd ratio	*p*‐value	95% CI	AUC	Cut‐off value
L3/4	−0.042	Per additional degree	0.959	0.002	0.934–0.984	0.666	53.11
L4/5	−0.101	Per additional degree	0.904	<0.001	0.874–0.934	0.937	53.14
L5/S1	−0.021	Per additional degree	0.979	0.017	0.961–0.997	0.606	82.78

Abbreviations: AUC, area under curve; CI, confidence interval; DLS, degenerative lumbar spondylolisthesis; FJA, facet joint angle.

### Short‐Term Follow‐up Results After Operation

The anteroposterior and lateral radiographs obtained at 2 days, 1 month, and 3 months after surgery showed no evidence of screw loosening, breakage, emerging endplate injury, or cage subsidence and migration. One patient was re‐admitted to the hospital because of surgical site infection 2 weeks after surgery, with fever as a clinical presentation. Infection was controlled after 6 weeks of antibiotic treatment and the infection did not cause the failure of internal fixation during the follow‐up.

## Discussion

A total of 125 adult patients with single‐segment spondylolisthesis who underwent retractor‐assisted TLIF were included in this retrospective study. All DLS occurred in L4, while only 39.0% of ILS occurred in L4. The cause of spondylolisthesis in L5 is typically the isthmus. Epidemiological studies^2^, ^22^ reported that the incidence of lumbar spondylolysis in the general adult population was 5.9%–6.4%, and the male‐to‐female ratio was about 2:1, 90% of which occurred in L5, while most of the remaining cases occurred in L4. This was likely because the isthmus at L5 level was easily affected by the direct clamp effect of the inferior articular process of L4 and the superior articular process of S1. Although the progression of spondylolisthesis in adults is much less than that in adolescence due to the ossification of the growth plate,^23^ it still exists, but the mechanism is still not clear. In a long‐term follow‐up study of 18 adults with spondylolysis, Floman^24^ reported that spondylolysis is most likely to progress in adults between the ages of 40 and 60 years, which may be associated with the reduced resistance of the degenerative disc to forward lumbar shear forces. A biomechanical study performed by Ramakrishna *et al*.^25^ showed that in the case of bilateral pedicle defects in L5, even mild degeneration will lead to an abnormal increase in the range of intervertebral motion and shear stress, and the reduction of DH is the mitigation mechanism for this change. A cross‐sectional study^2^ showed that although more than half of the patients with spondylolysis progressed to spondylolisthesis, most patients had no severe symptoms requiring surgery. The incidence of DLS in the adult population is approximately 6%, mostly in the age range of 61–75 years, among which the incidence in L4 is approximately four times higher than that in L5.^26^ Moreover, the incidence in women is about three to six times higher than that in men.^26^, ^27^, ^28^ In this study, most DLS were of low grade, and none of the patients with DLS had an SD of more than 30%. The preoperative SL (5.98 ± 1.90 mm *vs* 8.37 ± 3.29 mm, *p* < 0.001) and SD (17.70 ± 5.62% *vs* 25.18 ± 9.73%, *p* < 0.001) in the DLS group were significantly smaller than those in the ILS group. However, both groups showed a similar degree of reduction after surgical treatment (5.91 ± 3.12% *vs* 7.16 ± 5.69%, *p* = 0.135).

### Key Technical Notes and Theoretical Advantages of Screw Retractor‐Assisted TLIF

Open TLIF can sufficiently decompress neuronal components and stabilize slippage segments, which has been proven to be an effective treatment for LS complicated with neurological symptoms and has been widely promoted in clinical applications. However, LS may be accompanied by narrowed intervertebral space, and such anatomical abnormalities are more common in middle‐aged and elderly patients. Therefore, multiple techniques have been developed to address this problem including “insert‐and‐rotate” and “distract‐and‐reduce” techniques. In the “insert‐and‐rotate” technique, the paddle‐like intervertebral distractor can expand the intervertebral space and complete partial spondylolisthesis reduction, which has been widely proven to be effective in long‐term applications.^11^, ^29^ On this basis, Li *et al*.^12^ modified the instrument, using the rolling effect of the “ball‐bone” interface to improve the lifting efficiency of the distractor.

Moreover, several instruments have been reported to improve the efficiency of the “distract‐and‐reduce” technique for spondylolisthesis. The main working principle is to apply a lever force on the adjacent screws, such as the SOCON® system^13^ and the polyaxial screw and rod fixation system proposed by Liu *et al*.^30^ Meanwhile, Tumialan *et al*.^14^ developed a provisional ipsilateral expandable rod for MIS‐TLIF to achieve stable intervertebral height recovery and coronal balance.

However, problems were found during clinical practice, lumbar displacement traction on the dura may result in an abnormal anatomical position of the nerve roots. For example, when the nerve roots descended to the posterior edge of the intervertebral disc due to the collapse of the intervertebral space (Figure 2A), it was difficult and dangerous to remove the disc directly with a sharp knife. In our series, we used a screw retractor to expand the intervertebral space properly, thereby stretching the folded ligamentum flavum, and also made the abnormally positioned nerve roots leave the posterior edge of the disc (Figure 2B), thus the spinal canal and nerve root could be fully seen at any time during decompression and reduction. The screw retractor is inspired by the Caspar pin retractor,^31^ which not only enables the combination of the two technologies, but also provides adequate visual field exposure and neuroprotection. We changed the sleeves into two L‐shaped connecting rods in opposite directions, which offered following advantages: (i) a wide range of applications that can be applied to almost all screw‐rod systems without additional customized locking nuts or sleeves; (ii) convenient installation and removal without additional surgical exposure; and (iii) gradual, effective, and stable distraction from the check ratchet.

We combined the “insert‐and‐rotate” and “distract‐and‐reduce” techniques to modify the TLIF with the assistance of a screw retractor (Figure 2C). The ligaments surrounding the vertebrae were stretched as the intervertebral space was gradually expanded using interbody distractors and screw retractors alternately; therefore, the spondylolisthesis was reduced immediately through ligament tension. Sears *et al*.^11^ reported that restoring DH can usually reduce the slip by approximately 50% in spondylolisthesis of I or II degree. A study by Lian *et al*.^19^ revealed that pre‐distraction can reduce the demand for reduction, and based on the findings of our study, the ideal reduction can be achieved by the back‐translation force generated by the in situ locking of rods and cranial screws (Figure 2D,E). This operation sequence of “distract‐reduce” allowed for the reduction of spondylolisthesis without relying entirely on screws lifting the vertebra. Based on previous experience, for elderly patients with osteoporosis, the holding force of screw lifting vertebra is weak, and direct lifting or distraction with instruments may cause screw loosening or pullout. The displacement of screw lifting was relatively small in our methods, which may be worth trying in cases of spondylolisthesis with osteoporosis.

In traditional interbody fusion techniques, rimers or cage models were used to expand the intervertebral space before cage implantation. Such direct expansion may damage the endplate, 22 of 235 patients underwent interbody fusion had endplate injury according to the study of Zeng *et al*.^18^ In our methods, we adopted the interbody distractors for progressively blunt expansion of the intervertebral space, five sizes of intervertebral distractors from 8 to 12 mm were selected generally. Each slight expansion was captured by the retractor's check ratchet in time to avoid excessive stress from the intervertebral distractor on the endplate. Furthermore, the locked retractor left enough space for cage insertion, which may reduce the damage caused by cage to the endplate during the implantation. Overconcentration of stress may occur when a single instrument is adopted, and the combination of the two instruments theoretically improves this situation. Although we have achieved good clinical outcomes, confirmation of its superiority still requires prospective control studies.

### Efficacy and Safety of Screw Retractor‐Assisted TLIF

Radiographic data before surgery showed that the DH of the ILS group was significantly lower than that of the DLS group (6.36 ± 2.70 mm *vs* 9.01 ± 1.71 mm, *p* < 0.001), which improved more (3.69 ± 1.97 mm *vs* 2.12 ± 1.19 mm, *p* < 0.001) after TLIF; however, it was still significantly lower than that of the DLS group (10.05 ± 1.91 mm *vs* 11.13 ± 1.36 mm, *p* < 0.05). For the cases enrolled in this study, the spondylolisthesis was significantly reduced, and the effect did not differ between the ILS and DLS groups. Moreover, DH, IA, and SA have been restored. These outcomes were sufficient in proving that TLIF assisted by screw retractor was effective.

We performed TLIF using a kidney‐shaped cage, which was inserted obliquely. The height restoration of the foramen on both sides was similar (2.84 ± 2.81 mm *vs* 3.06 ± 2.96 mm, *p* > 0.05). Radiographic results in the short term after surgery indicated that single‐cage TLIF assisted by a unilateral retractor did not cause significant differences in the morphology between bilateral foramina. The screw retractor provided temporary distraction during surgery; however, the most crucial operation for long‐term maintenance of coronal balance is placing the kidney‐shaped cage in the center of the intervertebral space. In our experience, the change of resistance can be felt when the cage reaches the center of the intervertebral space. After unloading the retractor, adjacent vertebral bodies clamping the cage guaranteed the coronal balance. Its safety and effectiveness have also been confirmed in previous studies, because single‐cage TLIF could achieve the same biomechanical stability similar to that of the double‐cage TLIF.^32^, ^33^ Furthermore, Lee *et al*.^34^ reported no significant difference in clinical and radiographic outcomes between patients who underwent single‐cage PLIF and double‐cage PLIF for spinal degenerative diseases. In addition, Kroppenstedt *et al*.^35^ verified that there was no significant difference between the two groups regarding the maintenance of spinal stability and cage subsidence after 8 years of follow‐up.

Moreover, the excessive expansion of the intervertebral space can be a risk for the long‐term existence of fusion elements. Pisano *et al*.^36^ emphasized that the excessive height of the interbody cage is an independent risk factor for postoperative cage subsidence. Le *et al*.^37^ recommended that the height range of the interbody cage used in TLIF should be 8–12 mm. Seuk *et al*.^38^ followed 105 patients with spondylolisthesis who underwent interbody fusion and found that lower DH and greater segmental lordosis were relevant to poor postoperative leg symptoms. It is recommended that the restoration of intervertebral height and good bony fusion should be regarded as a more important surgical goal in treating spondylolisthesis, rather than the excessive restoration of segmental lordosis or slip correction.

However, conclusions based on short‐term results still have some limitations. Hence, we will conduct a long‐term follow‐up in the future to explore the following questions: (i) Can lumbar vertebrae maintain long‐term balance on the coronal plane? and (ii) Will single‐cage TLIF increase the risk of complications including cage subsidence, cage migration, and iatrogenic lumbar scoliosis?

### Correlations between FJAs and DLS

Bilateral facet joints and corresponding segmental intervertebral discs form a “three‐joint complex,” which plays an important role in the activity and load‐bearing of the spine. In normal individuals, the orientation of the facet joint of the upper lumbar vertebrae tends to the sagittal plane, while the facet joint of the lower lumbar vertebrae is more inclined to the coronal plane, which makes the lower lumbar vertebrae bear more weight. We investigated the characteristics of the FJA from L3/4 to L5/S1 and found that the FJA of L4/5 was significantly smaller than that of the L3/4 and L5/S1. Wang *et al*.^39^ reported the variation characteristics of FJA of “large‐small‐large” in patients with DLS, which is consistent with the results of this study. Logistic regression analysis showed that the FJAs in the lower lumbar spine were highly correlated with the occurrence of DLS, indicating that a smaller FJA was a risk factor for DLS. In addition, the cut‐off value of L4/5 FJA was 53.14°, which means that patients with FJA less than 53.14° were more likely to have DLS (AUC = 0.937). Guo *et al*.^40^ selected the angle formed by the tangent between the unilateral facet joint surface and the posterior edge of the vertebral body to represent the orientation of the facet joint and proposed that patients with angles greater than 60.19° were more likely to have DLS. Although the measured angles were different, the conclusions were similar.

On the cross‐section, we observed a decrease in the FJAs of patients with degenerative spondylolisthesis, which was considered to be the pathogenic factor for DLS. Previous biomechanical studies^41^ have suggested that the sagittal facet joint increases the shear force of the lumbar spine, and this pre‐existing morphological change may be a risk factor for the occurrence or progression of LS. In contrast, some researchers believe that degenerated discs change the kinematics of the mobile segments, and that compensatory hypertrophy and morphological changes of facet joints are the result of degenerative remodeling instead of the cause.^27^ Previous studies have focused on the morphology of facet joints in the slipped segment but ignored the facet joints in healthy segments.^41^ In this study, we found that the angles of facet joints in each segment of the lower lumbar vertebrae in the DLS group were smaller than those in the control group, which may indicate that there were morphological characteristics of facet sagittalization before the onset of DLS. In the DLS group, the long‐term result of the increase of the anterior shear force may be facet subluxation, which leads to degenerative remodeling changes such as hyperplastic periostosis and hypertrophy of the facet joint. This also explains the “big‐small‐big” change in the angle from the L3/4 to L5/S1 facet joint. For patients who undergo radiographic examination because of back pain, physicians could offer a forward‐looking advice based on the FJAs to prevent the occurrence or progression of DLS to some extent. By all means, the current results are still insufficient to clarify the true effect of facet orientation on the pathogenesis or progression of spondylolisthesis. Prospective studies with higher levels of evidence to demonstrate causality are still required.

### Strengths and Limitations

This was a retrospective study that described the pedicle screw retractor. The retractor has the following advantages: (i) it fits almost all pedicle screws and is convenient to install and remove; (ii) it provides more space to operate and protects the nerves in discectomy; (iii) the retractor had the capacity to maintain the disc height achieved by paddle distractors, allowing for the progressive and stable distraction of the intervertebral space by exchanging distractors. This study demonstrated that this technique did not cause the endplate damage or coronal imbalance.

Nevertheless, the limitations include the enrolment of only patients with single‐segment spondylolisthesis. Further studies evaluating the scope of application of the pedicle screw retractor in multi‐segment spondylolisthesis and other spinal diseases, including lumbar disc herniation, spinal deformities, and spinal tumors, must be considered. Limited by the fact that we only reviewed short‐term postoperative radiographic outcomes, parameters such as pain, neurological function, and quality of life were not studied regretfully, requiring us to conduct prospective studies in the future with long follow‐up to fully evaluate the technique and to identify complications such as cage subsidence and fusion failure that were neglected during short‐term follow‐up.

## Conclusion

The TLIF with unilateral screw retractor brought significant radiographic corrections on vertebral displacement, disc height, foramen height, and segmental lordosis in adult patients with low‐grade single‐segmental spondylolisthesis. Short‐term radiographic outcomes have shown that surgical reduction was sufficient and stable, with no significant complications such as endplate injury, screw loosening, or iatrogenic scoliosis, demonstrating that this instrument was safe and effective. Patients with facet sagittalization were more likely to have degenerative spondylolisthesis, while the cut‐off angle of L4/5 for predicting L4 spondylolisthesis was 53.19.

## Author Contributions

All authors had full access to the data in the study and take responsibility for the integrity of the data and the accuracy of the data analysis. Conceptualization—Hua Zhang and Hongwei Xie; Methodology—Hua Zhang and Hongwei Xie; Investigation—Hongwei Xie and Ziyu Ouyang; Formal Analysis—Hongwei Xie and Ziyu Ouyang; Writing of Original Draft—Hongwei Xie; Writing (Review and Editing)—Hua Zhang, Hongwei Xei, and Ziyu Ouyang; Visualization—Hongwei Xei; Supervision—Hua Zhang; Funding Acquisition—Hua Zhang.

## Conflict of Interest

The authors declare no conflicts of interest.

## Ethics Statement

The study was approved by the Ethics Committee for Human Research in Second Affiliated Hospital of Zhejiang University School of Medicine (Reference Number: 0424) and was in compliance with the Declaration of Helsinki.
